# *NRG1* fusions in breast cancer

**DOI:** 10.1186/s13058-020-01377-5

**Published:** 2021-01-07

**Authors:** Karen D. Howarth, Tashfina Mirza, Susanna L. Cooke, Suet-Feung Chin, Jessica C. Pole, Ernest Turro, Matthew D. Eldridge, Raquel Manzano Garcia, Oscar M. Rueda, Chris Boursnell, Jean E. Abraham, Carlos Caldas, Paul A. W. Edwards

**Affiliations:** 1 Hutchison-MRC Research Centre, University of Cambridge, Cambridge, CB2 0XZ UK; 2 Department of Pathology, University of Cambridge, Cambridge, UK; 3 Present addresses: Inivata Ltd, Babraham Research Park, Cambridge, CB22 3FH UK; 4 Present addresses: Francis Crick Institute, Midland Road, London, NW1 1AT UK; 5 Present addresses: Wolfson Wohl Cancer Research Centre, Garscube Estate, Bearsden, G61 1QH UK; 6 Department of Oncology, Cancer Research UK Cambridge Institute and Cancer Centre, Li Ka Shing Centre, University of Cambridge, Cambridge, CB2 0RE UK; 7 Present addresses: Illumina Cambridge, Granta Park, Great Abington, Cambridge, CB21 6GP UK; 8 Department of Haematology, University of Cambridge, Cambridge Biomedical Campus, Cambridge, CB2 0PT UK; 9 Present addresses: Mindich Child Health and Development Institute, Icahn School of Medicine at Mount Sinai, New York, NY 10029 USA; 10 Present addresses: MRC Biostatistics Unit, University of Cambridge, Cambridge Biomedical Campus, Cambridge, CB2 0SR UK; 11 Cambridge Breast Cancer Research Unit, NIHR Cambridge Biomedical Research Centre and Cambridge Experimental Cancer Medicine Centre at Cambridge University Hospitals NHS Foundation Trust, Cambridge, CB2 2QQ UK

**Keywords:** NRG1 fusions, Breast cancer, NRG1, Clinically actionable, MDA-MB-175, Whole-genome sequencing, Transcriptome sequencing

## Abstract

**Background:**

*NRG1* gene fusions may be clinically actionable, since cancers carrying the fusion transcripts can be sensitive to tyrosine kinase inhibitors. The *NRG1* gene encodes ligands for the HER2(ERBB2)-ERBB3 heterodimeric receptor tyrosine kinase, and the gene fusions are thought to lead to autocrine stimulation of the receptor. The *NRG1* fusion expressed in the breast cancer cell line MDA-MB-175 serves as a model example of such fusions, showing the proposed autocrine loop and exceptional drug sensitivity. However, its structure has not been properly characterised, its oncogenic activity has not been fully explained, and there is limited data on such fusions in breast cancer.

**Methods:**

We analysed genomic rearrangements and transcripts of *NRG1* in MDA-MB-175 and a panel of 571 breast cancers.

**Results:**

We found that the MDA-MB-175 fusion—originally reported as a *DOC4(TENM4)-NRG1* fusion, lacking the cytoplasmic tail of *NRG1*—is in reality a double fusion, *PPP6R3-TENM4-NRG1*, producing multiple transcripts, some of which include the cytoplasmic tail. We hypothesise that many *NRG1* fusions may be oncogenic not for lacking the cytoplasmic domain but because they do not encode NRG1’s nuclear-localised form. The fusion in MDA-MB-175 is the result of a very complex genomic rearrangement, which we partially characterised, that creates additional expressed gene fusions, *RSF1-TENM4*, *TPCN2-RSF1*, and *MRPL48-GAB2*.

We searched for *NRG1* rearrangements in 571 breast cancers subjected to genome sequencing and transcriptome sequencing and found four cases (0.7%) with fusions, *WRN-NRG1*, *FAM91A1-NRG1*, *ARHGEF39-NRG1*, and *ZNF704-NRG1*, all splicing into *NRG1* at the same exon as in MDA-MB-175. However, the *WRN-NRG1* and *ARHGEF39-NRG1* fusions were out of frame. We identified rearrangements of *NRG1* in many more (8% of) cases that seemed more likely to inactivate than to create activating fusions, or whose outcome could not be predicted because they were complex, or both. This is not surprising because *NRG1* can be pro-apoptotic and is *in*activated in some breast cancers.

**Conclusions:**

Our results highlight the complexity of rearrangements of *NRG1* in breast cancers and confirm that some do not activate but inactivate. Careful interpretation of *NRG1* rearrangements will therefore be necessary for appropriate patient management.

## Introduction

The *NRG1* gene encodes ligands for the ERBB2(HER2)-ERBB3 heterodimeric receptor tyrosine kinase [1–3]. Gene fusions of *NRG1* such as *CD74-NRG1* and *SLC33A2-NRG1* have been found at low frequency in a wide range of carcinomas including lung, breast, colorectal, ovarian, and pancreatic cancers and with a wide range of fusion partners ([4–9], reviewed in [10]). Although the frequency is generally low, 0.2% overall in a wide range of carcinomas according to Jonna et al. [9], it rises to roughly 25% in the rare invasive mucinous adenocarcinoma of the lung [6].

The fusions that retain the EGF-like receptor-binding domain are thought to be activating and oncogenic by creating an autocrine loop in which the fused NRG1 protein stimulates the heterodimeric ERBB2-ERBB3 receptor [3, 7, 11, 12]. Such cancers might be very sensitive to inhibition of ERBB2-ERBB3 [13], and there are several recent reports of good responses in patients to anti-ERBB2 or anti-ERBB3 therapy, including anti-ERBB3 antibody and HER-family kinase inhibitors such as the pan-ERBB inhibitor afatinib. This has led to the proposal that *NRG1* fusions are clinically actionable [7, 8, 14–18] and, as a result, NRG1 fusions have been included in the TAPUR study matching patients that have driver mutations to appropriate therapy (Clinical trials ref. NCT02693535).

The *NRG1* fusion of the breast cancer cell line MDA-MB-175 [11, 19, 20] was the first *NRG1* fusion reported and serves as a model of such fusions and the proposed autocrine loop. MDA-MB-175 cells secrete a fused NRG1 protein that was originally thought to be an isoform of NRG1 [11] but subsequently was reported to be a DOC4 (now renamed TENM4)-NRG1 fusion [19, 20]. The cells secrete a fusion protein that stimulates ERBB3 phosphorylation when added to other cells [13], and they are very sensitive to tyrosine kinase inhibitors, being the cancer cell line most sensitive to a dual ERBB2-ERBB3 inhibitor in the survey of Wilson et al. [13].

However, the structure of this *NRG1* fusion has not been completely described. It was reported to be a *DOC4/TENM4-NRG1* fusion, but the original cDNA sequence [11] is in fact a double fusion *PPP6R3-TENM4-NRG1* implying a complex genomic rearrangement. Furthermore, normal *NRG1* has many splice variants: the original fusion cDNA—and indeed many of the fusion transcripts described in clinical samples—represents only one of potentially many isoforms. In particular, the original cDNA lacked the cytoplasmic tail, and this has been hypothesised to enhance its oncogenic activity, since the cytoplasmic tail has been linked to pro-apoptotic activity of *NRG1* [21] (see also the “Discussion” section).

We report here the full transcript structure with alternatively spliced variants, partial characterisation of the underlying complex genomic rearrangement, and other gene fusions from the same genomic regions that presumably result from the same complex rearrangement. We also report a search for *NRG1* fusions and rearrangements in nearly 600 breast cancers.

## Methods

MDA-MB-175 (ATCC catalogue HTB-25) was obtained from the collection of Dr. M. J. O’Hare, Ludwig Cancer Research (who also provided the immortalised normal breast cell line HB4A [22]), and is the same stock as we used for karyotyping [23]. Its authenticity is confirmed by the presence of the fusion. It was maintained in DMEM Glutamax +15% FBS.

Genome positions are given relative to hg19/GRCh37 unless otherwise marked.

Paired-end transcriptome sequencing (RNA-seq) for MDA-MB-175 used Illumina’s stranded RNA kit with polyA selection. Forty-one million mapped reads were obtained after removing duplicates and were analysed with TopHat-Fusion [24]. Additional cell line transcriptome (RNA-seq) data was downloaded from the Cancer Genome Atlas (TCGA) project, using the Cancer Genomics Hub (now superseded by www.cancer.gov) as mapped sequence reads.

Genomic DNA sequencing of MDA-MB-175: DNA was captured by hybridisation using Nextera Custom Target Enrichment kit (Illumina, Great Chesterford, UK). Nextera uses a modified Tn5 transposase to simultaneously fragment DNA and attach a transposon sequence to both ends of the fragments. Fragments were PCR amplified and barcoded in 11 cycles of PCR; quantified using Qubit HS dsDNA assay (Life Technologies, CA.) and 500 ng pooled into a pool of twelve samples. 80-mer enrichment probes were designed by Illumina to *NRG1* genomic regions, from hg19/chr8:31696790-31873798 and 32140458-32310458, both within intron 1, at intermediate probe density, and 32320000-32500000, at dense probe density, 85 kb upstream of exon 2 to just beyond exon 6. Capture was performed twice to increase specificity. Enriched libraries were amplified using universal primers in 11 cycles of PCR, their quality assessed using Bioanalyser (Agilent Technologies, Ca.) and quantified using KAPA Library Quantification Kits (Kapa Biosystems, Ma.). Four capture reactions (48 samples) were pooled for 125-bp paired-end sequencing in a lane of Illumina HiSeq 2000. Structural variants were called as described [25] and calls manually inspected using the IGV (Integrative Genomics Viewer). Copy number was estimated from read counts using geneCN [26, 27].

### Breast cancers

Data was from consecutive consented patients with a successful DNA extraction, in the Cambridge Personalised Breast Cancer Program, led by JEA and CC, to be described elsewhere. Eighty-eight percent tumours were primary, 12% metastatic at sampling (Supplementary Tables 3 and 4 give details). DNA and RNA from tumours and DNA from matched blood were paired-end sequenced by Illumina, Great Chesterford, UK, using respectively the TruSeq® DNA PCR-Free Library Preparation kit or the TruSeq® Stranded Total RNA Library Preparation Gold kit. For DNA, reads were 150 bp to minimum 75X, typically 100X, coverage of tumour after alignment and removal of duplicates; for matched normal minimum 30X, typically near 40X. RNA sequencing was approximately 100 million 75-bp read pairs per tumour, but without removal of duplicates. Structural variants and copy number aberrations were called by Illumina using Manta [28] following alignment with Isaac [29] to GRCh38 with decoy sequences. Structural variants were further filtered to remove calls with any supporting reads in the matched normal, calls also found in the pooled matched blood normal samples, and calls involving unassembled or mitochondrial chromosomes [25].

Fusion transcripts were identified in individual RNA reads by text searching the original sequence (fastq) files for *NRG1* splice acceptor and splice donor sequences and extracting adjacent sequences. DNA structural variant calls were available for 250 patients of which 235 had RNA sequencing; RNA sequences from a further 336 cases were searched for additional fusions and, in the two cases identified, the matching DNA sequences were analysed individually for rearrangements.

## Results

### The *NRG1* fusion in MDA-MB-175 is a double fusion *PPP6R3-TENM4-NRG1*

**Fig. 1:**
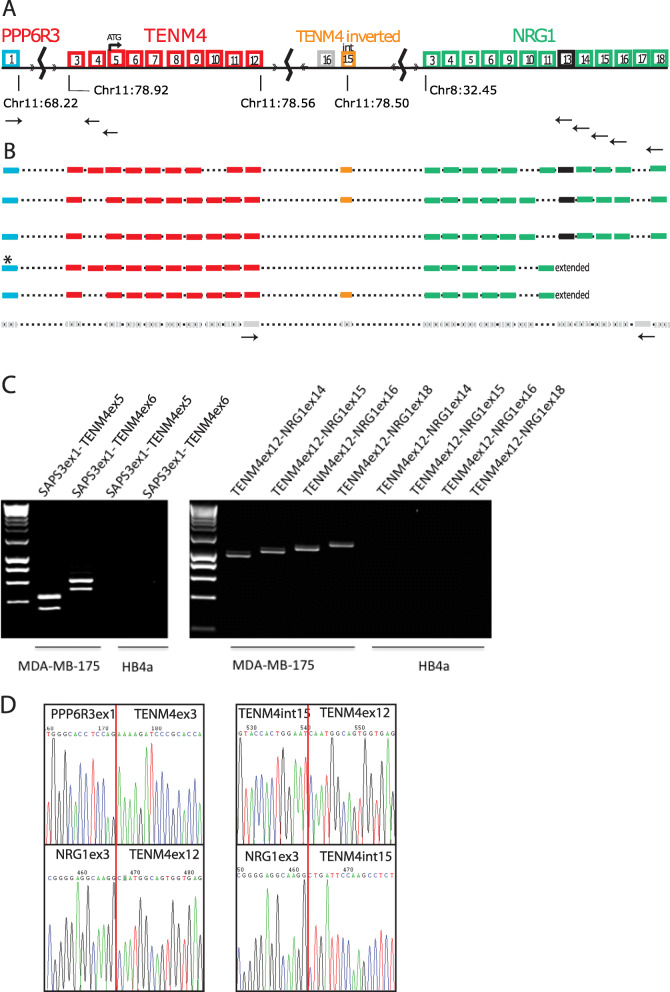
Structure of *PPP6R3-TENM4-NRG1* fusion transcripts expressed by MDA-MB-175. **a** Schematic of genes involved, showing hypothetical genomic structure deduced from the transcripts. Exons from *PPP6R3* are shown in blue, *TENM4* exons, red, *NRG1* exons, green, with the transmembrane domain exon in black. Cryptic exon from *TENM4* intron 15 in reverse orientation, orange. In grey, possible position of exon 16 of *TENM4* for orientation, not present in fusion transcripts. *NRG1* exons are numbered according to genome position. Exon 9 is the receptor-binding domain; exons 3 and 4 are the Ig-like domain. Exons 1, 2, 7, and 8 are omitted because they are normal transcription start sites and do not participate in the fusion. Where transcription terminates in exon 11, the exon is extended to give the β3 isoforms. Genomic positions are hg19. **b** Transcripts detected. In colour, transcripts amplified by PCR and cloned; grey, additional isoform that includes exon 17, inferred from successful amplification between primers shown. Asterisk marks transcript matching original cDNA of Schaefer et al. [11]. **c** Examples of confirmation of junctions by PCR, showing inclusion of cytoplasmic exons of *NRG1* in fusion. Primer pairs are shown by arrows in **a**. HB4a is control normal breast cell line. **d** Sequences through junctions

The *NRG1* fusion transcript of breast cancer cell line MDA-MB-175 was originally described as a fusion of *DOC4* (now *TENM4*, encoding teneurin transmembrane protein 4, also called *ODZ4*) to *NRG1* [11, 19, 20]. However, the original cDNA sequence Genbank AF009227 [11] is in fact a double fusion, 5′ *PPP6R3-TENM4-NRG1* 3′ (Supplementary Table 1; Fig. 1). The fusion cDNA comprises the first, non-coding exon of *PPP6R3* (protein phosphatase 6, regulatory subunit 3; formerly SAPS3), correctly spliced to exons 3 to 12 of *TENM4*, and then to exon 3 of *NRG1* (exons numbered according to their order in the genome, exon 3 is the second exon of many normal *NRG1* transcripts; Fig. 1, Supplementary Table 2). The *PPP6R3-TENM4* (formerly *SAPS3-ODZ4*) cDNA junction was previously detected by Robinson et al. (Supplementary data of ref. [30]) in transcriptome sequencing, but recorded as a separate fusion.

We confirmed the double-fusion structure, first by RT-PCR and Sanger sequencing (Fig. 1 c, d) and paired-end sequencing of cDNA fragments (‘RNA-seq’) (Supplementary Figure 1) and finally by amplifying, cloning, and Sanger sequencing complete cDNAs extending from exon 1 of *PPP6R3* through the *TENM4* component to various exons of *NRG1* (Fig. 1; Supplementary Textfile 1).

### The fusion extends to the 3′ end of *NRG1*, including the cytoplasmic tail

*NRG1* has many alternative isoforms [1]. Although the original cDNA, AF009227 [11], lacks the cytoplasmic exons 12 to 18, terminating in exon 11ext (Fig. 1; Supplementary Tables 1, 2), which defines normal β3 isoforms such as Heregulin-β3 (RefSeq NM_013958) and *NRG1* Type III-β3, we detected expression in MDA-MB-175 of all the later exons as well, in fusion transcripts (Fig. 1). We amplified complete cDNAs extending from *PPP6R3* exon 1 to the last *NRG1* exon, exon 18, which on cloning included at least three isoforms: we detected both the alpha and beta forms of *NRG1* (respectively including *NRG1* exon 10 or 11), and two transcripts included an additional, unannotated exon we designated ‘exon int15’. This exon is in reverse orientation within intron 15 of *TENM4*, at hg19/chr11:78,506,385-78,506,462 (hg38 chr11:78795340-78795417), and therefore must be transcribed from an inverted fragment of chromosome 11 inserted into the main *TENM4-NRG1* junction (Fig. 1; Supplementary Table 2; Supplementary Textfile 1). RNAseq confirmed the presence of this exon (Supplementary Figure 2 panel C), but showed it was a minor variant, as only 24% (38/160) of split reads across the junction with *NRG1* exon 3 were from this exon (the others were all from *TENM4* exon 12). This exon would insert 26 amino acids and preserve the reading frame downstream.

We also amplified the originally reported transcript that terminates in an extension of exon 11 and a variant that included the inverted exon int15 (Fig. 1; Supplementary Textfile 1).

### Other gene fusion transcripts: *RSF1*-*TENM4*, *TPCN2*-*RSF1*, *MRPL48*-*GAB2*

RNAseq revealed other cDNA junctions in MDA-MB-175 from further gene fusions caused by related rearrangements within chromosome 11, and we confirmed them by RT-PCR: *RSF1* exon 3 fused to exon 15 of *TENM4*, TPCN2 exon 5 fused to *RSF1* exon 4, and *MRPL48* exon 3 fused to *GAB2* exon 2 (Supplementary Figures 3, 4). A normal transcript of *RSF1*, exons 1 to 5, was also detected by PCR, and RNAseq showed expression of all *RSF1* exons with normal splicing, the apparently normal expression exceeding that of the fusions.

### Expression of *NRG1*, *TENM4*, and *PPP6R3* other than in the fusion

*NRG1*, *TENM4*, and *PPP6R3* are all expressed in normal breast, *PPP6R3* strongly, *NRG1* and *TENM4* weakly (GTEx RNAseq database accessed via UCSC Genome browser and [31]). We asked whether there was expression of unrearranged *NRG1*, *TENM4*, and *PPP6R3* in MDA-MB-175 or of a hypothetical precursor fusion, *PPP6R3-TENM4* or *TENM4-NRG1*, a copy of which might still be present. For *NRG1*, no expression was detected from the major transcription start sites of *NRG1* exon1, exon 2, and exon 7, either by RT-PCR or transcriptome sequencing (RNA-seq; (Supplementary Figure 2)), and all splicing into *NRG1* exon 3 was from *TENM4* exons 12 or int15 (total 160 split reads). *NRG1* exon 2 is the main transcription start site in normal breast epithelial cells and carcinoma cell lines [31]. We previously showed that in MDA-MB-175, the CpG island at exon 2 is methylated [31]. However, a recently described alternative minor transcription start site, exon 8, found in isoform ndf43c/VI-1 (Refseq NM_001159996 and NM_001322197) was weakly expressed (Supplementary Figures 1 and 2), and we amplified a cDNA extending from exon 8 to exon 18, comprising exons 8 to 10 and 13 to 18, and a second cDNA with exon 17 omitted. We presume these are normal transcripts, since splicing into exon 8 has not been described, and PCR failed to amplify between *TENM4* and exon 8 of *NRG1*.

Similarly, almost no expression was detected from *TENM4* exons 1 and 2 (Supplementary Figures 1 and 2; manual search found 5 split reads joining exons 1 and 2), although data from the Cancer Genome Atlas (TCGA) shows that these are expressed in other breast cancer cell lines such as MCF7 and MDA-MB-134, and we failed to amplify cDNA joining exon 2 of *TENM4* to *NRG1*. By RNAseq, only 1 of 145 split reads that included the splice acceptor of *TENM4* exon 3 matched exon 2 splice donor; all others were consistent with splicing in from *PPP6R3* (two included an alternative exon 2 of *PPP6R3* and two reads included intronic sequence from upstream of exon 3 in *TENM4*). This seems to rule out significant expression of both normal *TENM4* and a *TENM4-NRG1* precursor. However, there was good expression of *TENM4* exons beyond the last exon involved in the fusion, exon 12, and splicing was normal, including exon 12–exon 13. Although some of this expression would be the *RSF1*-*TENM4* fusion (Supplementary Figures 1 and 2), this only starts at *TENM4* exon 15 and there is splicing exon14 to exon 15, and no other splicing anomalies were detected: a *PPP6R3*-*TENM4* fusion may therefore remain. We failed to amplify between *PPP6R3* exon 1 and *TENM4* exon 16, but this might have been technical failure.

Expression of *PPP6R3* exons not in the fusion was also evident, presumably from a normal copy (Supplementary Figure 1).

### Genome rearrangements associated with the fusions

We were able to demonstrate some of the complexity of the underlying genomic rearrangements that formed the *PPP6R3-TENM4-NRG1* fusion, by constructing a plausible, though incomplete, model of them from the gene fusions, data from FISH (fluorescence in situ hybridisation), paired-end sequencing of part of the *NRG1* region, and copy number.

**Fig. 2:**
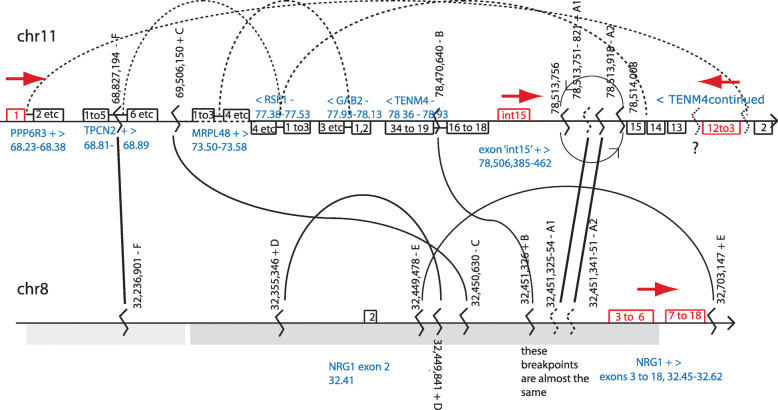
Partial genomic structure of rearrangements in MDA-MB-175 from capture sequencing and RNA fusions. Regions capture-sequenced shown as grey shading: lighter grey, 32.14–32.310 Mb, intermediate probe density; darker grey, 32.320–32.5 Mb, denser probes. All positions hg19. Solid arcs, breakpoint joins from capture sequencing, with positions of breakpoints given in bp, with direction of join and a one-letter junction identifier, e.g. on chr8, junction marked ‘32,236,901 - F’, is junction F at 32,236,901 bp, ‘-’ indicating that sequence to the right of junction is retained. Dotted arcs, possible junctions in unsequenced regions, deduced from observed gene fusion transcripts. Blue, genes with their locations in Mb and +ve or −ve strand; numbered exons shown above line for + strand genes, below for − strand genes. Red, exons in fusion transcripts; red arrows, direction of transcription of fusion. Junctions on chromosome 8 correspond to the major copy number steps shown by the capture sequencing of this region of chromosome 8 (Supplementary Figure 6)

The fusion cDNA suggests that it was formed by a large inversion of chromosome 11 that joined *PPP6R3* to *TENM4* and a translocation joining chromosome 11 to chromosome 8 upstream of exon 3 of *NRG1*, with an additional local inversion that allows exon int15 to be included in some transcripts (Fig. 2).

Cytogenetically, a typical metaphase of MDA-MB-175 has two copies of an unbalanced 8;11 translocation, plus apparently normal chromosomes 8 (three copies) and 11 (two copies) [19, 23]. FISH with BAC clones showed that, as expected, the 8;11 translocation chromosome harbours all the three major genomic segments that form the fusion, apparently in more than one copy, with the *PPP6R3*, *TENM4*, and *NRG1* regions colocalised, while the normal chromosomes retain single copies (Supplementary Figure 5).

We identified a number of rearrangement junctions in genomic DNA that are consistent with the fusions, by paired-end sequencing of DNA captured by hybridisation from around exons 2 to 6 of *NRG1* (hg19/chr8: 31.7 to 31.874 and 32.14 to 32.50 Mb). This identified junctions within chromosome 8 and between this region and chromosome 11 (Fig. 2). These were curated manually using the Integrative Genomics Viewer (IGV), revealing an additional small inversion on chromosome 11 apparently encompassing the 8-11 junction that forms the fusion. Genomic copy number analysis by counting reads from the capture-sequencing (Supplementary Figure 6) confirmed the presence of unbalanced junctions corresponding to the rearrangement junctions in the region captured: up at junction marked F in Fig. 2, down at junction D, and up at the cluster of breakpoints marked A1, A2, B, C, and E.

The rearrangements shown are only part of a more complex picture. Array-CGH (array-comparative genomic hybridization) from [32] suggests multiple copy number steps in the rearranged regions (Supplementary Figure 6), and the additional fusions found suggest additional rearrangements, shown as dotted lines in Fig. 2. Our capture sequencing would not have found junctions that did not include the captured *NRG1* region—notably the *TENM4-RSF1* and *PPP6R3-TENM4* junctions.

#### Overexpression of the fusion

We transfected, into HEK293 cells, FLAG-tagged coding sequences of the principal isoforms of the fusion, with and without the cytoplasmic tail. FLAG-tagged protein of the expected size was detected on harvesting cells at 48 h (Supplementary Figure 7), showing that such isoforms can be expressed at least transiently. We also transfected isoforms that included the extra inverted exon int15, but these were not detectably expressed.

### *NRG1* fusions in primary breast cancers

To put the MDA-MB-175 fusion in the context of breast cancer, we surveyed 571 consecutive consented cases of breast cancer subjected to both whole-genome DNA and RNA sequencing. We identified four *NRG1* fusions of the form (*geneA*)-*NRG1*, that were both predicted from DNA rearrangement junctions and found in RNA sequence reads: *WRN-NRG1*, *FAM91A1-NRG1*, *ARHGEF39-NRG1*, and *ZNF704-NRG1* (Supplementary Table 3, which also gives tumour subtypes and other known driver mutations). However, while the *FAM91A1-NRG1* fusion was in frame, the *WRN-NRG1* and *ARHGEF39-NRG1* fusions were not. The *ZNF704-NRG1* fusion was to an undocumented exon in *ZNF704*, so its reading frame is unknown, but the fused sequence was in frame with *NRG1*.

All four fusions spliced into *NRG1* at exon 3, as in MDA-MB175, and many of the fusions described by others; no fusions that spliced into exons 4 to 9 (the receptor-binding exon) were detected. All were created by internal rearrangement of chromosome 8 (Supplementary Table 3). A *WRN-NRG1* fusion has been described before—*WRN* is the gene immediately 5′ to *NRG1* so the fusion is typically formed by genomic deletion—but the previous example included no *WRN* coding sequence, so would presumably have resulted in expression of NRG1 protein [5, 9]. A fusion of *ZNF704* has also been reported, but to *MYC*, in the lung [5].

Detecting these fusions was not straightforward. They were not called from the RNA sequences by the fusion detection software STAR-fusion [33], because of insufficient read coverage—indeed, in two of four cases, our RNA sequencing yielded only one or two split fusion reads (Supplementary Table 3). In addition, the *ZNF704-NRG1* fusion spliced from an undocumented exon so would not have been found using software that only considered known exons. The prediction of the *ZNF704*-*NRG1* fusion was tentative because the genomic rearrangement is complex. A plausible reconstruction (Supplementary Figure 8) was that in addition to the *ZNF704*-*NRG1* junction, there was a tandem duplication of about 57 kb of *NRG1*, encompassing the unused exon 7, with insertion of 24 kb of inverted sequence into the duplication junction.

A further 20 of the first 250 breast cancer cases had breakpoints within *NRG1* by DNA sequencing, 13 of which had multiple breakpoints, which would make fusion prediction difficult (Supplementary Table 4). No fusion transcripts were detected in the matching RNA sequencing, but depth of sequencing might have been limiting.

The short-read RNA sequencing did not enable us to determine whether these fusions included the cytoplasmic tail exons. Expression of these exons was detected (Supplementary Table 3)—clearly in two cases, *FAM91A1-NRG1* and *ARHGEF39-NRG1* but not conclusively in the other two cases where there were too few reads overall—but we could not tell whether these reads were from fusion transcripts or normal transcripts, from tumour or normal cells.

## Discussion

We have shown that the *NRG1* fusion of MDA-MB-175 is more complex than previously described, being a double fusion *PPP6R3*-*TENM4*-*NRG1* with multiple alternative transcripts, some including the cytoplasmic tail, and it is the result of complex genomic rearrangements. We also confirmed that similar fusions—coding sequence of another gene splicing into genomic exon 3 of *NRG1—*are found in breast cancers, supporting the use of this fusion as a model example. The structure of these fusions has implications for clinical identification of *NRG1* fusions, for understanding the subcellular location and secretion of NRG1 fusion proteins, and explanations of their oncogenicity.

### Identifying *NRG1* fusions is challenging

Our search for *NRG1* fusions in breast cases, and the complexity of the fusion in MDA-MB-175, illustrate that identifying *NRG1* fusions in clinical cases is not straightforward. We needed both the DNA and RNA sequencing to detect the fusions in cancers: there were too few supporting reads in the RNA sequencing to call them from RNA alone, while prediction from the DNA rearrangements alone would only have been provisional, particularly in our 14 examples where there were multiple breaks in *NRG1*. MDA-MB-175 itself is a case in point: with 7 breakpoints called within *NRG1* (Fig. 2), prediction of a fusion would have been very difficult and uncertain. Although we found 4 examples in 571 cases (0.7%), in rough agreement with the 2/120 found by Kim et al. [34] but a substantially higher prevalence than others [8, 9], there might well have been more.

The importance of correct interpretation is underlined by the probability that some *NRG1* rearrangements—including presumably the out-of-frame fusions—are *in*activating events as discussed below. Probably our [35] and others’ [36] estimates of around 5% of breast cancers having breaks within *NRG1* by FISH includes many cases where there is no fusion. In conclusion (as noted before [6, 8–10]), RNA analysis is probably necessary, and combining with DNA sequencing improves sensitivity and specificity, but, even with both, sensitive identification of fusions is challenging.

### Structure of the MDA-MB-175 fusion

**Fig. 3:**
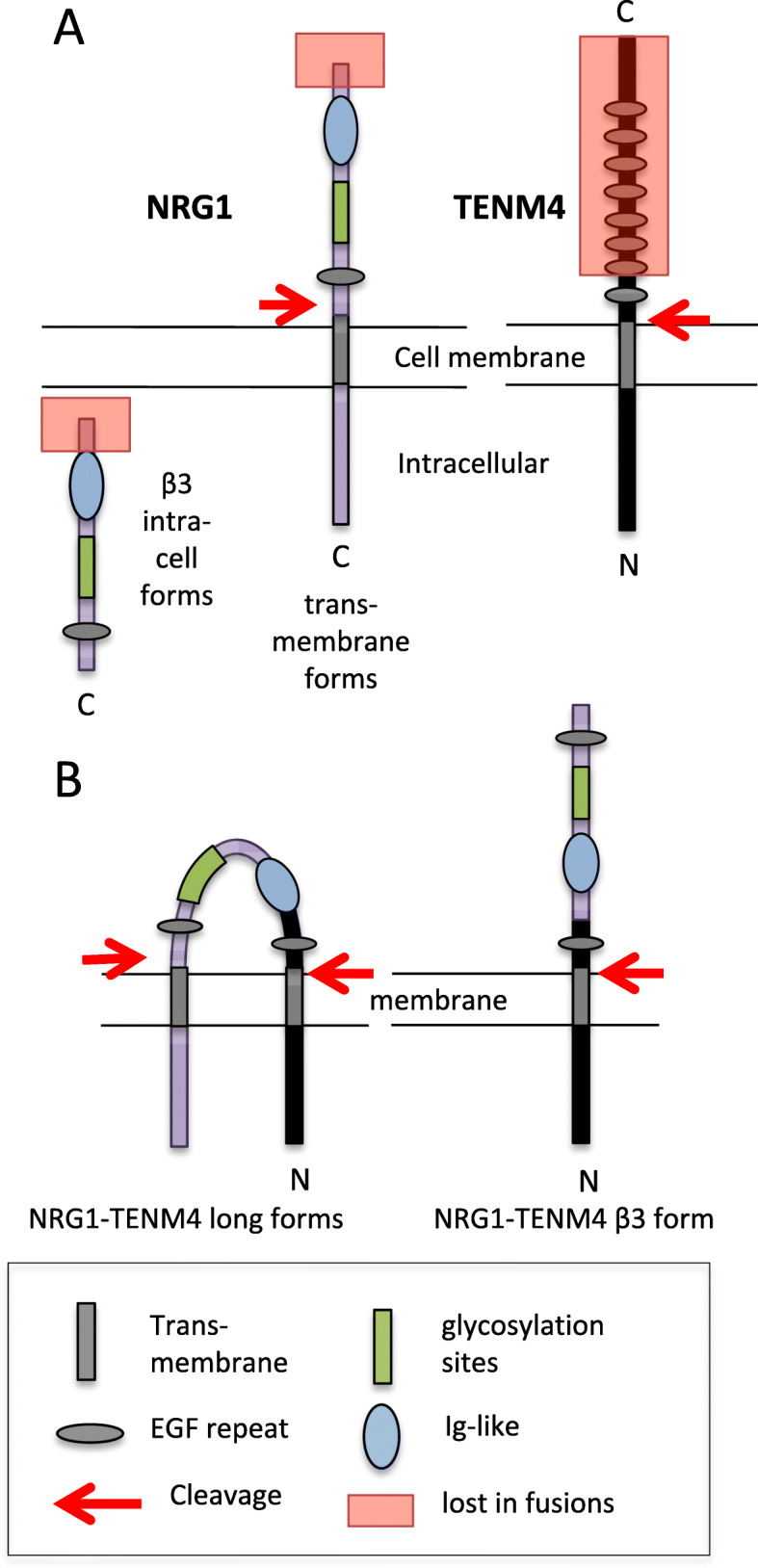
Predicted domain structure of the proteins encoded by the PPP6R3-TENM4-NRG1 fusion. Schematic diagrams showing the major domains detected by SMART [38]. **a** Normal structures: a typical transmembrane NRG1 and a β3 isoform, and TENM4 (*PPP6R3* contributes only untranslated sequence). At least some β3 isoforms go to the nucleus [1, 43]. **b** Predicted structure of fusion proteins. One EGF-repeat and the transmembrane domain of TENM4 are retained, juxtaposed to the Ig-like domain of NRG1. The chimeric protein may either include the transmembrane and cytoplasmic region of NRG1 or the short β3 terminus

The fusion partners *TENM4* and *PPP6R3* have not been seen in *NRG1* fusions in tissue samples, but this is not surprising, because there are already upwards of 30 known fusion partners (e.g. [9]). *TENM4*, teneurin4, has been identified as a probable driver target of structural variation, notably in the breast [37] and Fig. 3b of ref. [39], and its relative *TENM1*/*ODZ1* was identified as an oncogene target of the mouse mammary tumour virus (MMTV) [40]. It is a transmembrane protein with a cytoplasmic N-terminus and a large extracellular domain, most of which is lost in the fusion (Fig. 3).

An important feature of the *PPPR3*-*TENM4*-*NRG1* fusion is that, paralleling wild-type *NRG1*, we found multiple isoforms, including isoforms with the cytoplasmic tail (Fig. 1). The original cDNA cloned by Schaefer et al. [11] lacked the cytoplasmic domain of *NRG1*, and it has often been assumed that this was a feature of *NRG1* fusions in general. The isoforms we found (Fig. 1) all had the Ig-like domain, and they included both alpha and beta forms (alternative exons 10 and 11). Some had the full transmembrane and cytoplasmic C terminus designated 1a and 2a forms [1] while others, including the original cDNA of Schaefer et al. [11], terminated in an extended exon 11, designated -β3.

### Alternative splicing of other *NRG1* fusions

Many fusions have been presented as lacking the C-terminal, cytoplasmic exons, and terminating in the β3, non-transmembrane terminus (genomic exon 11ext). But the multiple splice forms in MDA-MB-175 suggests that these other fusions will also come in multiple isoforms, including forms with the cytoplasmic tail. Their absence from the literature is probably an oversight: partly a legacy of the original reports [4, 11] and partly technical, because short-read sequencing only shows the fusion junction, not downstream splicing patterns, and PCR or single-primer amplification of cDNA has often used primers in the β3 terminus (extended exon 11) or the EGF-like domain (exon 9) (e.g. [5, 9]).

Further confusion arises because some *NRG1* fusions have been described as derivatives of *NRG1* TypeIII-β3, but this is misleading: no fusions involve the transcription start site, genomic exon 7, that defines TypeIII neuregulins/heregulins, and many of the fusions include the Ig domains which are not in TypeIII-β3 [1].

It has also been assumed that the form of NRG1 secreted into the medium by MDA-MB-175 is encoded by the original cDNA of Schaefer et al. [11], but this may not be correct—it might be a cleaved fragment of a transmembrane isoform (Fig. 3).

### Oncogenic function of *NRG1* fusions is paradoxical

The oncogenic function of *NRG1* fusions is paradoxical and remains to be fully explained. The fusions apparently form an autocrine loop, stimulating the co-expressed ERBB-ERBB2/HER2 heterodimer [12, 13]. But normal epithelia produce both NRG1 and its receptors [31, 41], so why would *NRG1* fusions be oncogenic? And *NRG1* expression is pro-apoptotic when cDNAs are transfected into cells, including the breast cancer cell line MCF7 [42].

A possible resolution of this puzzle would be that NRG1 and its ERBB-family receptors are, in normal epithelium, produced by different cells, and/or on different faces of the cell [41], with co-expression in the same cell prevented by strong controls—perhaps leading to the apoptotic activity of transfected *NRG1* [42].

So why are *NRG1* fusions oncogenic? One previous hypothesis was that the cytoplasmic domain of *NRG1* is pro-apoptotic and is absent from the *PPP6R3*-*TENM4*-*NRG1* fusion [21]; our analysis rules this out.

We suggest two alternative explanations: alteration of expression or alteration of subcellular localisation. Simplest would be altered regulation of *NRG1* expression, by placing it downstream of an unrelated promoter, allowing one cell to express ligand and receptor. This would be consistent with the wide range of fusion partners.

### Loss of nuclear signalling?

A more intriguing hypothesis is that the fusion proteins have a different subcellular distribution, and, specifically, that one route of nuclear signalling is lost.

*NRG1* encodes many isoforms and proteolytically cleaved forms, secreted, membrane-bound, cytoplasmic, and nuclear [1, 43]. Among these, two entirely unrelated forms can signal to the nucleus: the cleaved cytoplasmic tail and the Type1-β3 form which includes the Ig-like and EGF-like domains (Fig. 3). The latter is intracellular because it lacks a transmembrane domain or signal sequence [2, 43], and it has been shown to translocate to the nucleus and alter gene expression [44, 45]. Translocation is mediated by sequences around the Ig-like domain [44, 46] (Breuleux et al. [46] used a truncated ‘heregulin-alpha’ cDNA that lacked a transmembrane domain).

The PPP6R3-TENM4-NRG1 fusion proteins consist of the intracellular part of TENM4 and its transmembrane domain, joined to a range of essentially intact NRG1 isoforms: the only exons of *NRG1* lost are the first two transcription-start exons (Fig. 3). Thus, TENM4 brings a transmembrane domain to the fusion, and the TypeI-β3 forms that would normally be intracellular presumably become extracellular (Fig. 3). Similarly, as noted by others (e.g. [5]), several fusion partners bring a transmembrane domain, including two of the commonest, *CD74* and *SLC3A2*. Other fusion partners have a signal sequence, e.g. *SDC4* [5], *CLU* [8], *ADAM9* [9], or fuse with loss of the Ig-like domain, e.g. some *CD74* fusions, *RBPMS*, *TSHZ2* [9], again denying β3 forms access to the nucleus. However, this is not a universal feature of the fusions, e.g. the *FOXA1* and *ROCK1* fusions [8].

### *NRG1* can be a tumour suppressor or oncogene

Although *NRG1* appears to be oncogenic in some tumours, it is *in*activated in carcinomas at least as often as it is activated. *NRG1* is silenced by methylation in some breast and other carcinomas [31, 47, 48] and seems to be at least one target of distal 8p loss, which is one of the most frequent large-scale events in carcinomas [49]. Many of the rearrangements in *NRG1* appear not to fuse the gene or create a fusion that lacks the EGF-like, receptor-binding domain, or are simply out of frame. Examples include a deletion in a breast cancer that removes the ligand-binding domain [50] and three further inactivating deletions [51]: fusions that retain only the 5′ end of NRG1, e.g. two described by Drilon et al. [8], and 3′ fusions that splice in at the transmembrane domain [51]. Of 16 NRG1 fusions found in TCGA RNA-seq data by Hu et al. [52], only 6 appeared to be in-frame fusions of 3′ NRG1 that included the EGF-like domain: four retained only the 5′ end, and most of the others appeared out-of-frame. Many of the rearrangements of *NRG1* that we found in breast cancers did not or were unlikely to create an activating fusion (Supplementary Table 4), including the two fusions that were out of frame.

This dual role could be because high ERBB3 activity can be achieved in two ways: either *NRG1* is inactivated to permit high ERBB3 activity in all cells or at both faces or to prevent *NRG1*’s pro-apoptotic activity [42] (which may be a manifestation of the same control) or *NRG1* can form an oncogenic autocrine loop, if control preventing co-expression can be broken. The lack of nuclear signalling by the Ig-like domain might be part of the control mechanism.

Whether or not this is the explanation, because many *NRG1* rearrangements seem to be inactivating, the correct identification of activating fusions may require care.

## Conclusions

We show here firstly that the *NRG1* fusion of the breast cancer cell line MDA-MB-175, which serves as a model *NRG1* fusion, is more complex than previously reported. It is a double fusion *PPP6R3-TENM4-NRG1*; is the result of a complex genomic rearrangement; and, like normal *NRG1*, is transcribed into multiple isoforms with different subcellular locations. This sheds new light on the mechanism of action of *NRG1* fusions. Secondly, we confirm that around 0.5% of breast cancers have *NRG1* fusions of this activating type, but many more cases have rearrangements of the *NRG1* gene that seem more likely to inactivate the gene or, as in MDA-MB-175, are too complex to interpret from DNA sequence alone. *NRG1* rearrangements will therefore require careful analysis and interpretation for appropriate patient management.

## Supplementary information

**Additional file 1: Supplementary Figure 1.** Sashimi plots of RNA sequencing of fusion in MDA-MB-175. **Supplementary Figure 2.** RNA expression levels in MDA-MB-175 by RNA-seq. **Supplementary Figure 3.** Additional gene fusions in MDA-MB-175. **Supplementary Figure 4.** Examples of verification by PCR of fusions in MDA-MB-175. **Supplementary Figure 5.** Genomic localisation of fusion in breast cancer cell line MDA-MB-175, by FISH. **Supplementary Figure 6.** Genomic copy number changes in MDA-MB-175. **Supplementary Figure 7.** Expression of NRG1 fusion proteins by transfection in HEK 293T cells. **Supplementary Figure 8.** Simplest reconstruction of NRG1 rearrangements that create the *ZNF704-NRG1* fusion.

**Additional file 2: Supplementary Table 1.** Match of fusion cDNA AF009227, originally reported by Schaefer et al. [11], to Reference genome. **Supplementary Table 2.** Exons of *PPP6R3*, *TENM4*, *NRG1*. **Supplementary Table 3.** Fusions of *NRG1* in 571 breast cancers. **Supplementary Table 4.** All *NRG1* rearrangements in first 250 breast cancers.

**Additional file 3: Supplementary Textfile 1.** Representative sequences of NRG1 fusion transcripts.

## Data Availability

Sequence data for MDA-MB-175 are available from the corresponding authors on reasonable request. The breast cancer data are not publicly available because of patient confidentiality, but application for access may be made jointly to CC Carlos.Caldas@cam.ac.uk and JEA ja344@medschl.cam.ac.uk.

## Abbreviations

RNA-seq: Short-read whole transcriptome RNA sequencing
BAC: Bacterial artificial chromosome
FISH: Fluorescence in situ hybridisation
