# Supplementary material for: NRG1 fusions in breast cancer
Source: Breast Cancer Res. 2021 Jan 7;23:3. doi: 10.1186/s13058-020-01377-5 (PMC7788813; doi:10.1186/s13058-020-01377-5)
Supplement: Supplementary file 1 — Additional file 1: Supplementary Figure 1. Sashimi plots of RNA sequencing of fusion in MDA-MB-175. Supplementary Figure 2. RNA expression levels in MDA-MB-175 by RNA-seq. Supplementary Figure 3. Additional gene fusions in MDA-MB-175. Supplementary Figure 4. Examples of verification by PCR of fusions in MDA-MB-175. Supplementary Figure 5. Genomic localisation of fusion in breast cancer cell line MDA-MB-175, by FISH. Supplementary Figure 6. Genomic copy number changes in MDA-MB-175. Supplementary Figure 7. Expression of NRG1 fusion proteins by transfection in HEK 293T cells. Supplementary Figure 8. Simplest reconstruction of NRG1 rearrangements that create the ZNF704-NRG1 fusion. [file 13058_2020_1377_MOESM1_ESM.pdf]

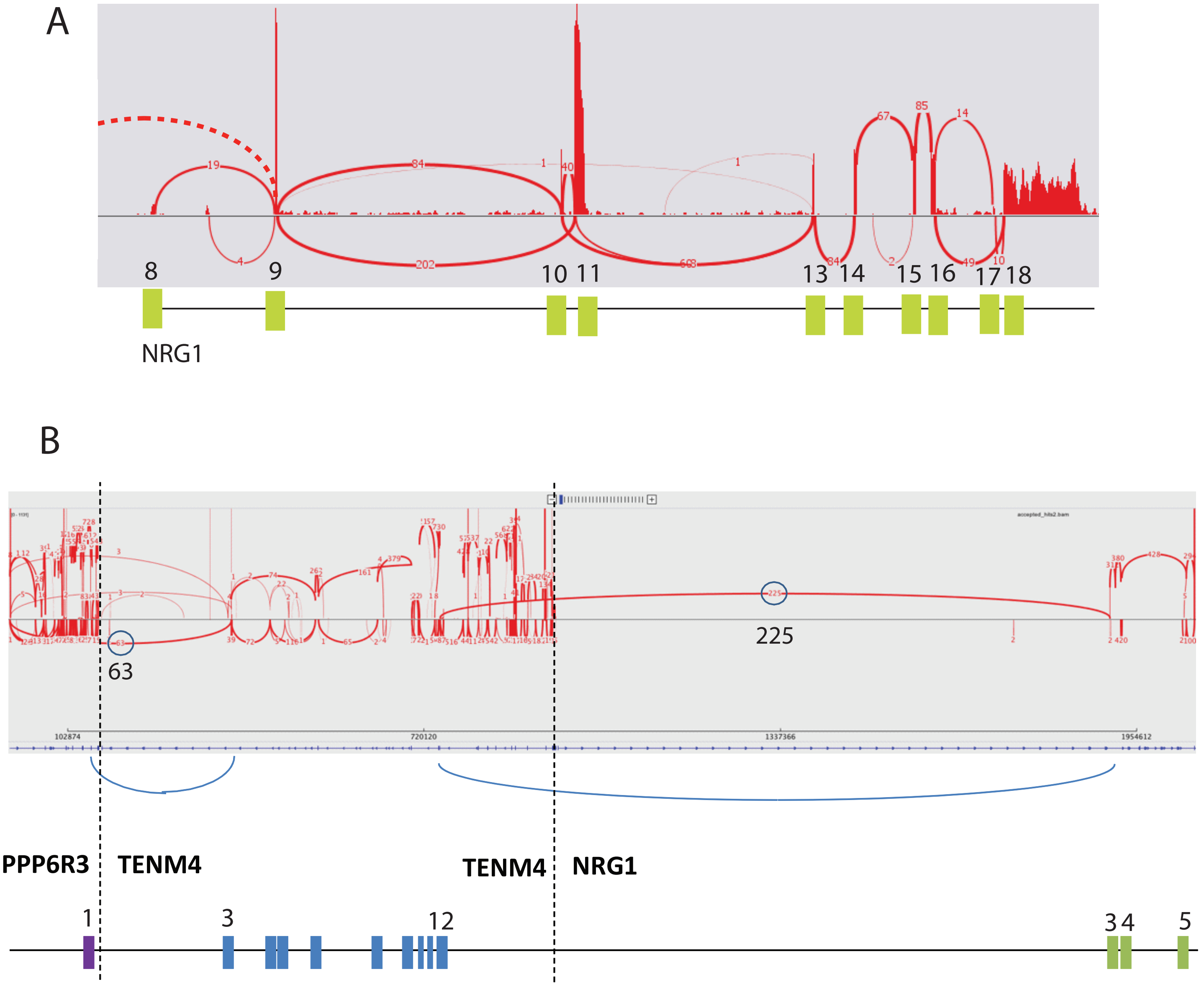

Supplementary Fig. 1. Sashimi plots of RNA sequencing. x-axes, genome position, with exons shown underneath; solid red peaks, reads per kilobase per million reads mapped (RPKM) within individual exons; arcs, reads that span different exons. A, reads from exons 8 to 18: these will be from fusion transcripts plus some normal expression from the minor normal transcription start site exon 8. Upstream exons were not included: splicing from exon 6 is represented by dotted arc. B. Larger region showing junctions PPP6R3-TENM4 and TENM4-NRG1 and ending at exon 5 of NRG1. Reads spanning the translocation junctions are highlighted with blue circles and arcs below, and the read depth is shown. Note absence of expression from exon 2 of NRG1. Expression of exons of PPP6R3 and TENM4 downstream of fusion junctions is presumably from normal copies of the genes or other rearrangments such as the RSF1-TENM4 fusion.

A

B

C

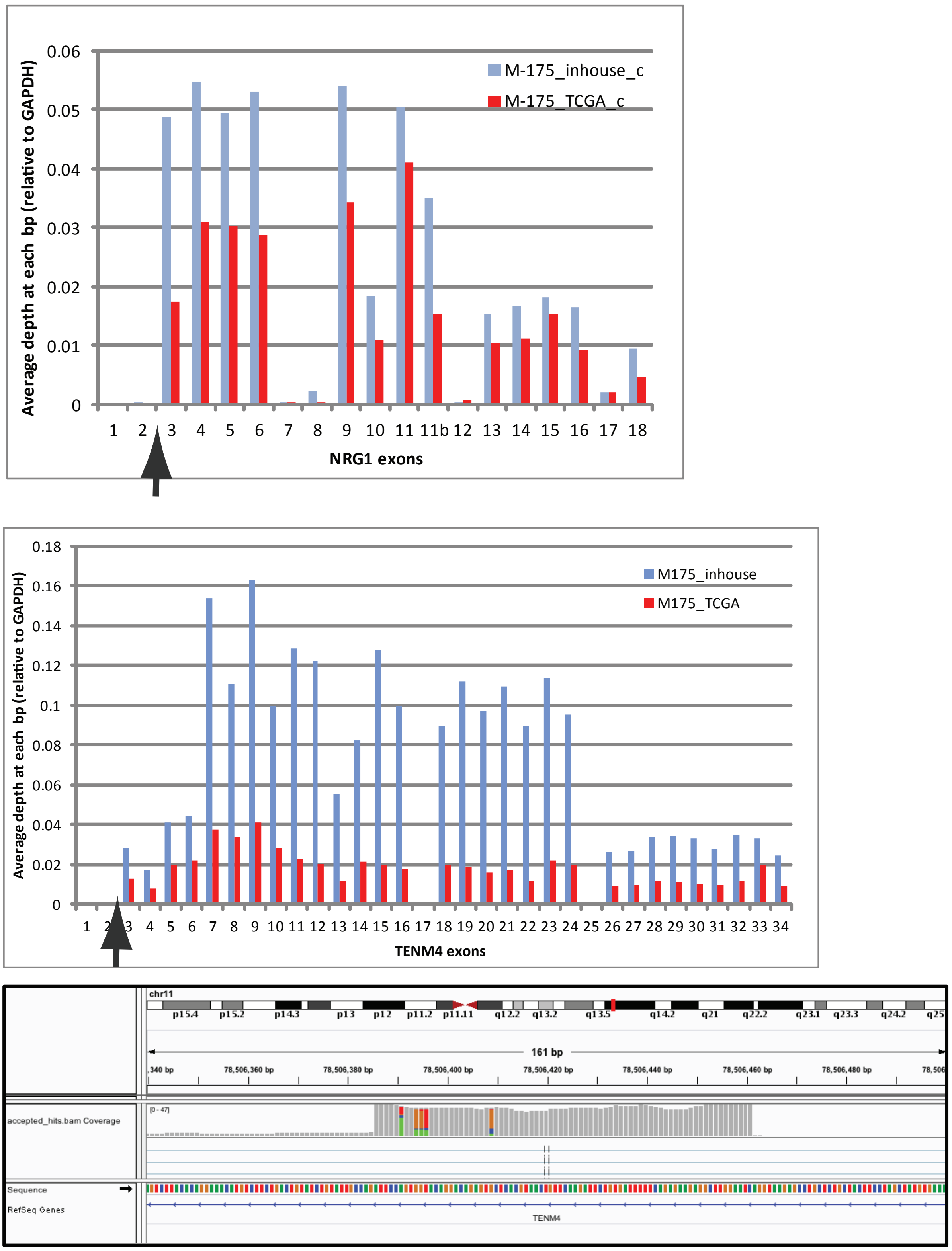

Supplementary Fig. 2. RNA expression in MDA-MB-175 by RNA-seq.
A. NRG1 expression. RNAseq data is displayed as average read depth per exon after correcting for exon size, relative to GAPDH. Blue bars, in-house RNAseq data; red bars, TCGA data. Arrow, breakpoint. Note absence of expression of exons 1 and 2. Exon 2 is the normal NRG1 start site in epithelia. There is no expression from exon 2 of the intact copy of NRG1 in this cell line probably due to methylation (Chua et al 2009 ref. 23). There is weak expression from alternative start site exon 8. B. TENM4 expression. Again, there is no expression of exons 1 and 2, although TCGA data shows that other breast cancer cell lines, such as MCF7 and MDA-MB-134, exress these exons. C. Cryptic exon in the reverse orientation in intron 15 of TENM4 that is spliced into some isoforms of the fusion. Read depth, displayed as grey bars in the Integrative Genomics Viewer, is about 8% of other TENM4 exons.

A

Fusion gene						
5’ partner	chr	3’ partner	chr	5’ position (hg19)	3’ position	Number of supporting reads (split reads; paired reads)
TENM4	11	NRG1	8	78565148	32453345	178 (149; 29)
TPCN2	11	RSF1	11	68825161	77451980	52 (36; 16)
RSF1	11	TENM4	11	77458080	78516536	40 (38; 2)
MRPL48	11	GAB2	11	73519394	77991946	13 (11; 2)
PPP6R3 (SAPS3)	11	TENM4	11	68228294	78926953	80 (52; 28)

B

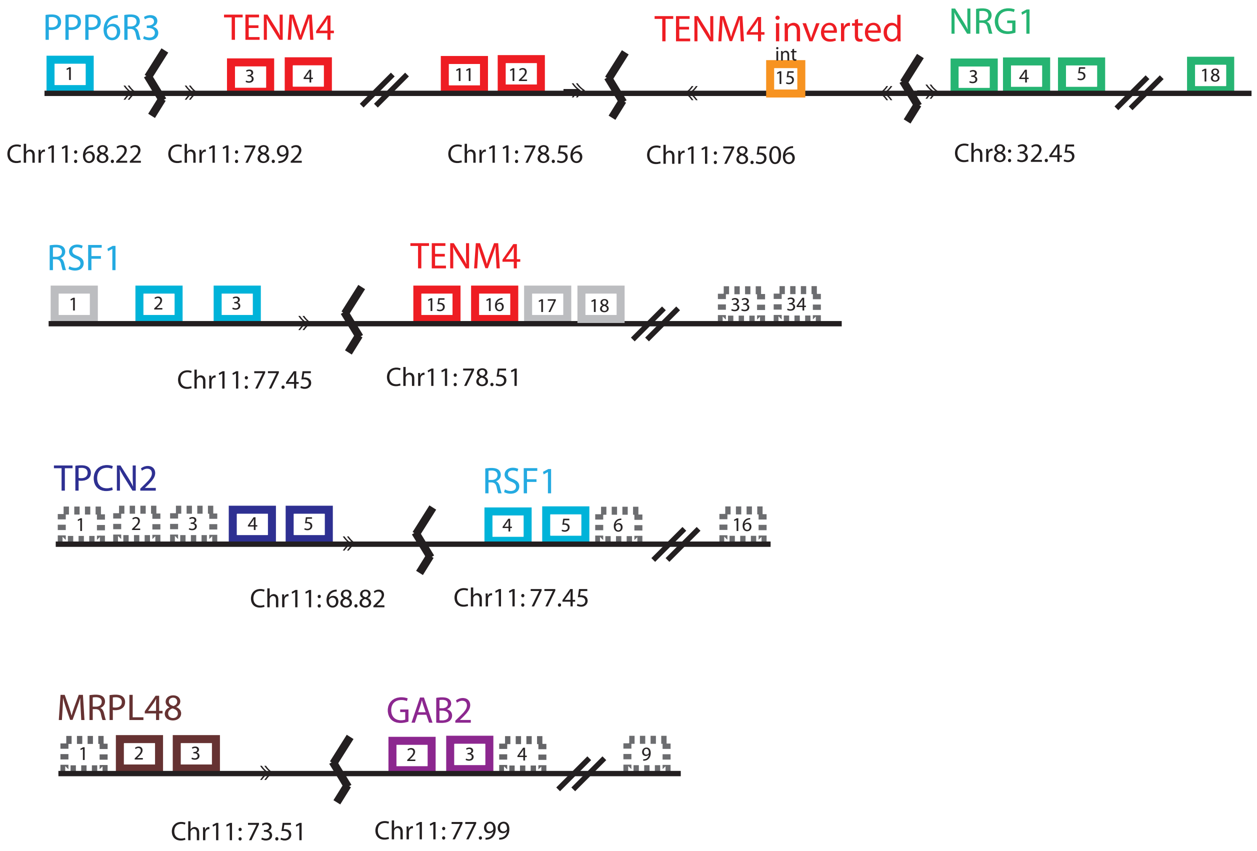

Supplementary Figure 3. Additional gene fusions. A, Fusion junctions detected by TopHat Fusion involving genes on chromosome 11, in RNA sequencing from MDA-MB-175. B, cDNA structures of the fusions, showing, in colour, exons detected by confirmatory RT-PCR using primer pairs that crossed the fusion junction. Exons shown in grey were not detected when PCR across the fusion junction was attempted; exons in dotted lines were not tested but are included for completeness. The alternative exon ‘int15’ in inverted intron 15 of *TENM4* was not called by TopHat Fusion but was found in some clones from the confirmatory PCR and present in the RNA sequences (Supplementary Fig. 2). Exon junction positions in Mb, hg19. *GAB2* exon numbering: exon 2 is second exon of both transcripts; would be 3rd exon in genome order.

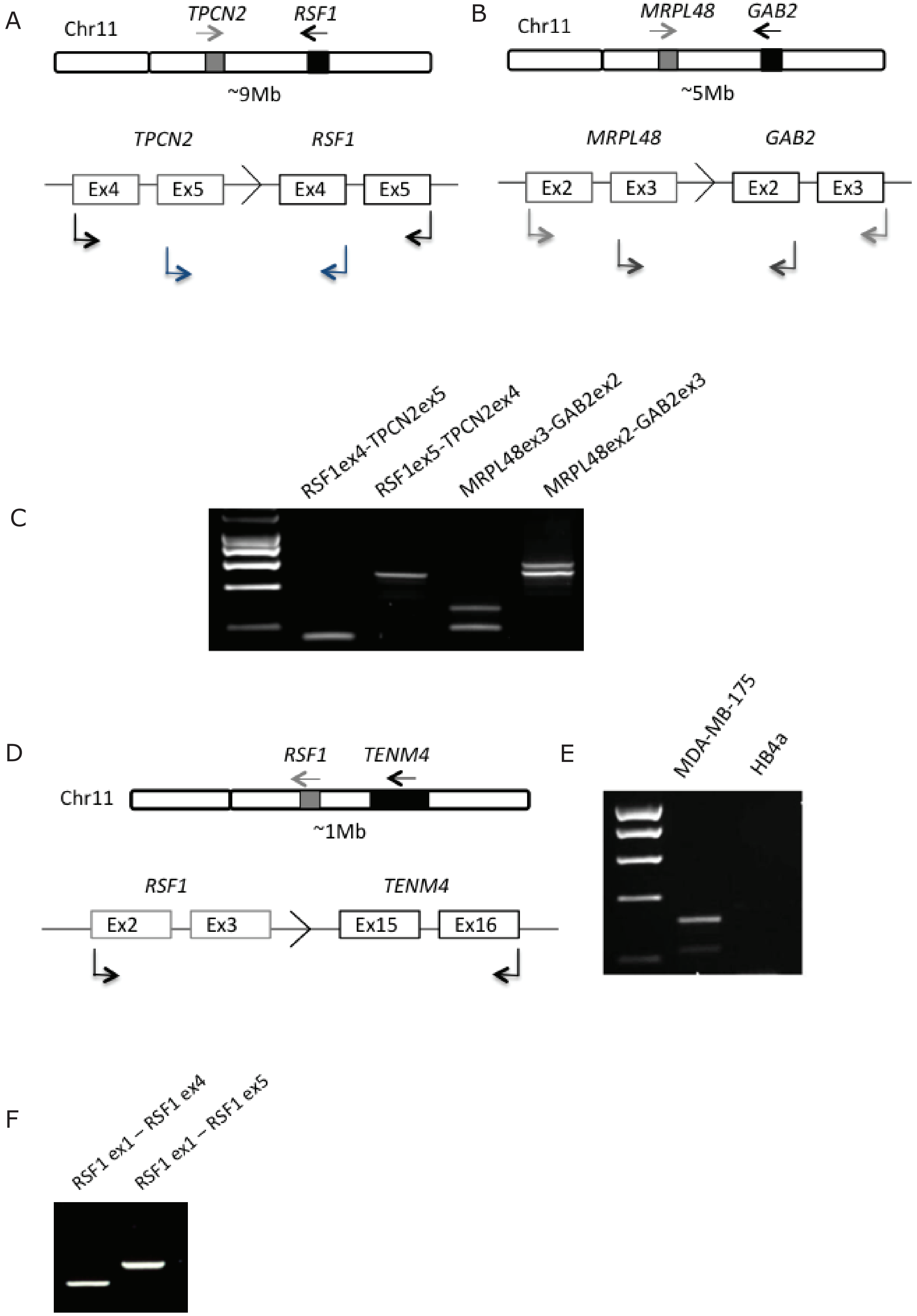

Supplementary Figure 4. Examples of fusion verification by PCR. A-C, TPCN2-RSF1 and MRPL48-GAB2 fusions. A,B schematic positions of genes on chromosome 11 and positions of PCR primers used to verify. C, PCR products, two primer sets for each fusion. D-F, TENM4-RSF1 fusion. E, successful amplification between RSF1 exon2 and TENM4 exon 16. F, Amplification of a normal RSF1 transcript spanning the two fusion junctions.

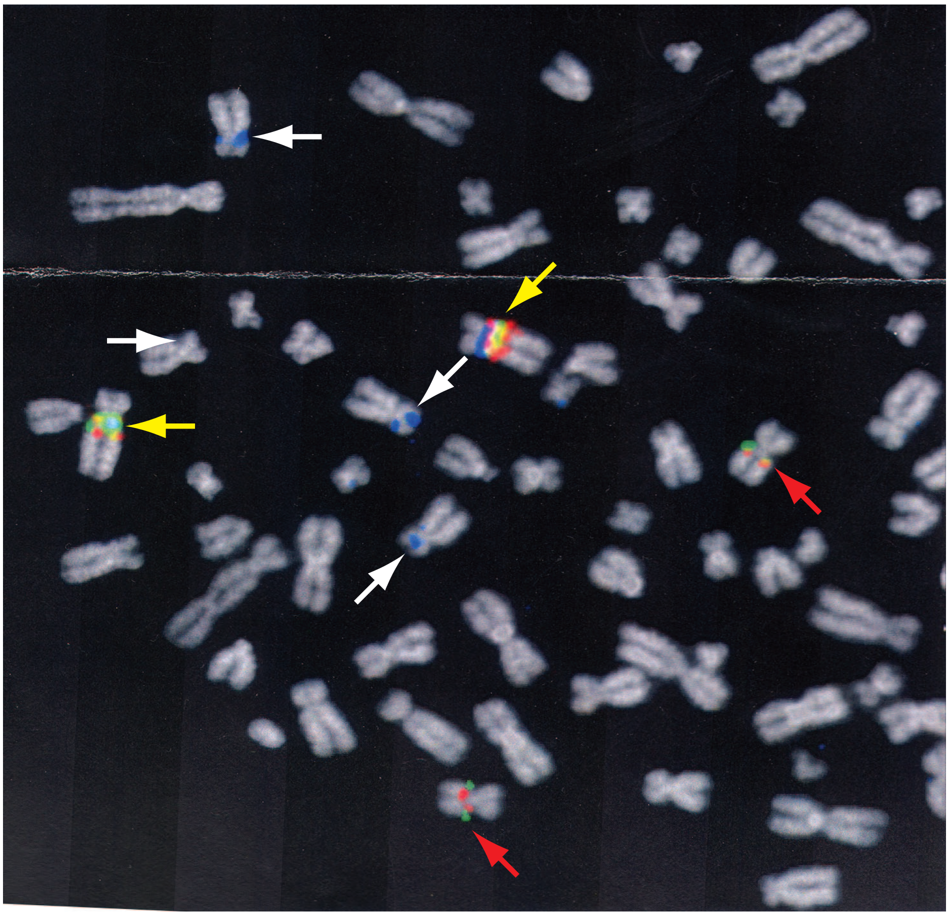

Supplementary Fig. 5. Genomic localisation of fusion in breast cancer cell line MDA-MB-175, by FISH with fluorescently-labelled BACs (methods described in Pole et al. Oncogene 2006:25,5693). Red signals, *TENM4*; green signals, *PPP6R3* (appears yellow or pale blue where superimposed on red or blue signals); blue signals, *NRG1*. Part of a metaphase spread showing two copies of the *PPP6R3-TENM4-NRG1* fusion (yellow arrows), with two normal copies of *PPP6R3* and *TENM4* on chromosome 11(red arrows) and three copies of *NRG1*on chromosome 8 (white arrows).

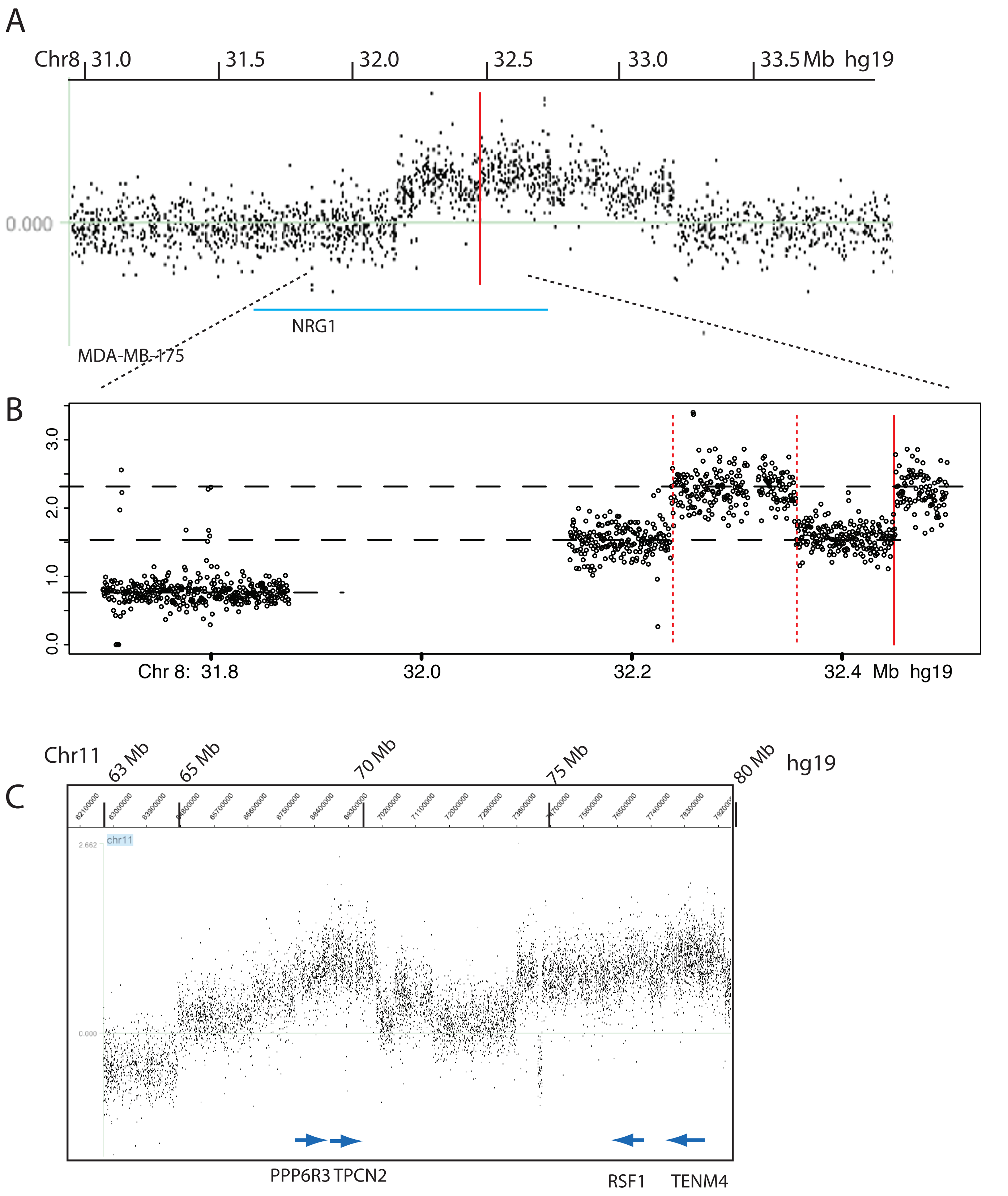

Supplementary Fig. 6. Genomic copy number changes in MDA-MB-175. A, chromosome 8, SNP6 array-CGH data from Bignell et al. Genome Research 2004;14: 287–95. Blue line, approximate location of NRG1; red line, breakpoint where NRG1 is joined to TENM4. B, higher-resolution copy number plot from read depth in capture sequencing, in 500 bp bins, divided by median number of reads in the bin over 96 tumour and cell line samples. Solid red line, breakpoint as in B, at 32.450 Mb, which matches cluster of junctions A1,A2,B,C,E in Fig. 2. Dotted red lines, copy number step down at 32.356 Mb corresponding to junction D in Fig. 2; and up at 32.237 Mb which corresponds to another 8;11 junction, junction F. Dashed horizontal lines are manually estimated copy number levels, which are close to ratio 1:2:3 and might correspond to 2,4 and 6 copies. C, chromosome 11, SNP6 array-CGH as in A. Blue arrows, approximate positions of genes PPP6R3 etc.

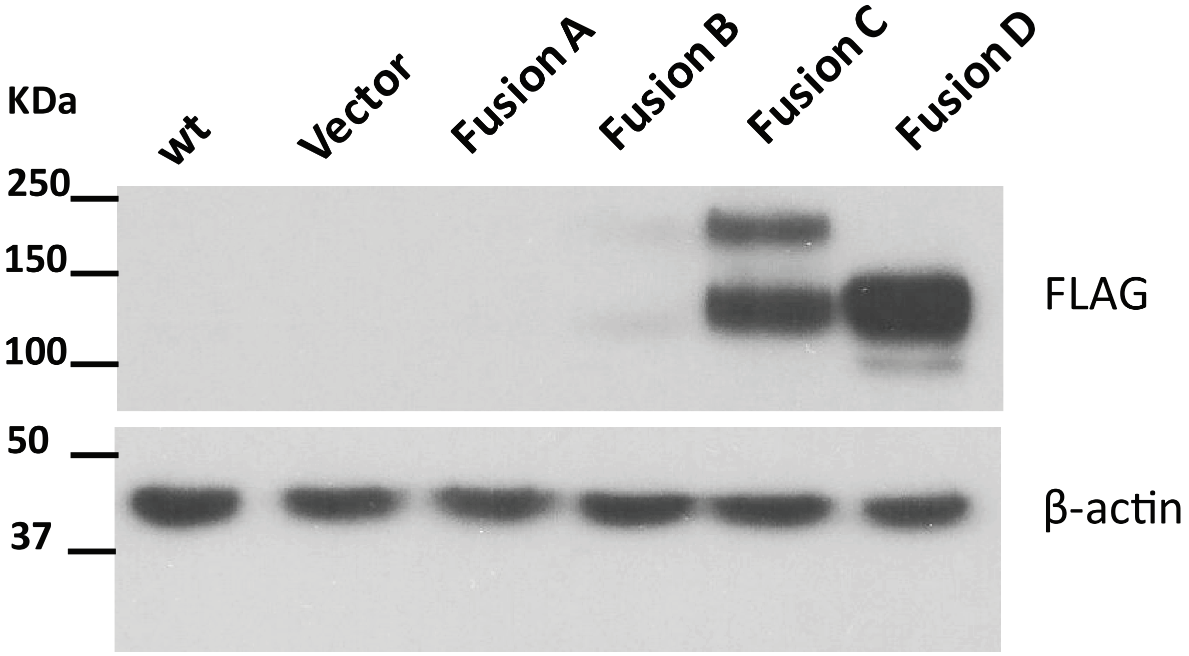

Supplementary Figure 7. Expression of NRG1 fusion proteins by transfection in HEK 293T cells.

cDNAs were amplified from the ATG in TENM4ex5 to the relevant NRG1 3'UTR and FLAG-tagged by cloning into pcDNA3-FLAG. Cell lysates (50μg protein) were made 48 hrs after transfection and probed with monoclonal antibody to FLAG (upper panel), and polyclonal to beta actin (lower panel).

wt, untransfected cells.

Fusion A (clone A1) – long isoform including intron 15 and exon 10

Fusion B (clone B3) – long isoform including intron 15 and exon 11

Fusion C (clone C5) – long isoform, no int 15, exon 10

Fusion D (clone D4) – as original cDNA of Schaefer et al: short isoform, terminating in extended exon 11 (beta3 isoform), no int 15.

Note that good expression was obtained only with clones that lack the 'extra' exon int 15, although the extra exon is predicted to retain reading frame.

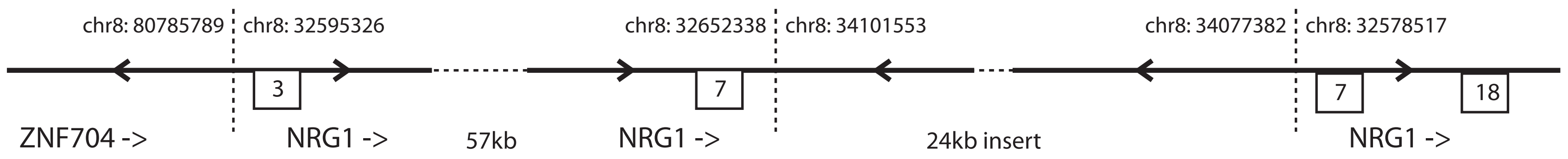

Supplementary Figure 8. Simplest reconstruction of NRG1 rearrangements that create the ZNF704-NRG1 fusion. Boxes with numbers are exons. Heavy lines represent genomic sequence, with the direction of the reference genome indicated by > . Genes are shown with direction of transcription. Dotted vertical lines represent breakpoint junctions detected by DNA sequencing; dotted horizontal lines represent hypothetical connections between the junctions. This model predicts that, in addition to the junction that fuses ZNF704 to NRG1, there is tandem duplication of part of NRG1 that includes the unused exon 7, with an insertion of 24kb of inverted genome into the duplication junction—but this duplication and insertion would have no obvious effect on the expressed fusion. Not to scale. Positions GRCh38/hg38
