# Supplementary material for: NRG1 fusions in breast cancer
Source: Breast Cancer Res. 2021 Jan 7;23:3. doi: 10.1186/s13058-020-01377-5 (PMC7788813; doi:10.1186/s13058-020-01377-5)
Supplement: Supplementary file 3 — Additional file 3: Supplementary Textfile 1. Representative sequences of NRG1 fusion transcripts. [file 13058_2020_1377_MOESM3_ESM.pdf]

Supplementary Textfile 1. Representative raw sequences from cloned full-length cDNA products.

Exon numbering according to their position in the genome, see Supplementary Table 2. The SNP in TENM4 exon 4 was also present in all of 55 RNAseq reads that covered the sequence. The clones for expression—cloned in-frame from the ATG in TENM4 exon5—were also sequenced to confirm the exons here.

Key:
Vector sequence
SAPS3 exon 1
TENM4 exons
TENM4 exon in opposite orientation ‘exon int15’
NRG1 exons
microhomology overlap
SNPs or errors in red

SAPS3-TENM4-NRG1 Clone D

Top transcript shown in Fig. 1. Long isoform with cytoplasmic tail, includes reversed exon TENM4 int15, lacks TENM4 exon 10

Sequencing with T7 primer:

vector
GGATCCACTAGTAACGGCCGCCAGTGTGCTGGA
ATTCTGCAGATATC
SAPS3 exon 1
TGCTTGATACGTCCGCCATTTTGGGCGCTTCGCTGATGGTGTCGGT
GAGCGCGTTTCCCGCCTGAGCGCAACTAGCGGCGGGTCGTGGGCACCTCCAG
TENM4 Exon3:
AAAAGATCCCGCACCATCCTCCAGGATCCAATGGCCTTGGAGAGAGGGCTGCAGGGCCCACGGACATT
GCTGACTCTTCAGAACGTGCTGACATGGAGCCAG
TENM4 Exon4: [T is a C in reference genome]
GTAGACTGAAATTATCATGTGTCCAAATTAAAATTGCATACTTCAAGGATTATTTGAAGGACTATTCTTAGACC
CTTTTAAGAAGATTTAAAGAAAA
TENM4 Exon5:
ACCACTCGGCCCTGAGTGCGGCGAGGACCCTGTTTGTGGATGTGGAGGA
GCGCGGGCCGGAGGCCATGGACGTGAAGGAGAGGAAGCCTTACCGCTCGCTGACCCGGCG
CCGCGACGCCGAGCGCCGCTACACCAGCTCGTCCGCGGACAGCGAGGAGGGCAAAGCCCC
GCAGAAATCGTACAGCTCCAGCGAGACCCTGAAGGCCTACGACCAGGACGCCCGCCTAGC
CTATGGCAGCCGCGTCAAGGACATTGTGCCGCAGGAGGCCGAGGAATTCTGCCGCACAG
TENM4 Exon6:
GTGCCAACTTCACCCTGCGGGAGCTGGGGCTGGAAGAAGTAACGCCCCCTCACGGGACCCT
GTACCGGACAGACATTGGCCTCCCCCACTGCGGCTACTCCATGGGGGCTGGCTCTGATGC
CGACATGGAGGCTGACACGGTGCTGTCCCCTGAGCACCCCGTGCGTCTGTGGGGCCGGAG
CACACGGTCAGGGCGCAGCTCCTGCCTGTCCAGCCGGGCCAATTCCAATCTCACACTCAC
CGACACCGAGCATGAAAACACTGAGACTG

Sequencing with TENM4exon5F:

CCGCCTAGCCTATGGCAGCCGCGTCAAGGACATTGTGCCGCAGGA
GGCCGAGGAATTCTGCCGCACAG
TENM4 Exon6:
GTGCCAACTTCACCCTGCGGGAGCTGGGGCTGGAAGA
AGTAACGCCCCCTCACGGGACCCTGTACCGGACAGACATTGGCCTCCCCCACTGCGGCTA
CTCCATGGGGGCTGGCTCTGATGCCGACATGGAGGCTGACACGGTGCTGTCCCCTGAGCA
CCCCGTGCGTCTGTGGGGCCGGAGCACACGGTCAGGGCGCAGCTCCTGCCTGTCCAGCCG
GGCCAATTCCAATCTCACACTCACCGACACCGAGCATGAAAACACTGAGACTG
TENM4 Exon7
ATCATCCGGGCGGCCTGCAGAACCACGCGCGGCTCCGGACGCCGCCGCCGCTCTCGCACGCCCA
CACCCCCAACCAGCACCACGCGGCCTCCATTAACTCCCTGAACCGGGGCAACTTCACGCC
GAGGAGCAACCCCAGCCCGGCCCCCACGGACCACTCGCTCTCCGGAGAGCCCCCTGCCGG
CGGCGCCCAGGAGCCTGCCCACGCCCAGGAGAACTGGCTGCTCAACAGCAACATCCCCCT
GGAGACCAG
TENM4 Exon8
AAACCTAGGCAAGCAGCCATTCCTAGGGACATTGCAGGACAACCTCATTGA
GATGGACATTCTCGGCGCCTCCCGCCATGATGGGGCTTACAGTGACGG
TENM4 Exon9
GCACTTCCTCTTCAAGCCTGGAGGCACCTCCCCGCTCTTCTGCACCACATCACCAGGGTACCCACTGACGTC
CAGCACAGTGTACTCTCCTCCGCCCCGACCCCTGCCCCGCAGCACCTTCGCCCGGCCGGC
CTTTAACCTCAAGAAGCCCTCCAAGTACTGTAACTGGAAGTGCGCAGCCCTGAGCGCCAT
CGTCATCTCAGCCACTCTGGTCATCCTGCTGGCATACTTTGTGG
TENM4 Exon11
GAAAGCCCAGTAGTTTCTTTCCAGAGGACAN TTTCATAGATTCTGGAGAAATTGATGTGGG

Sequencing with NRG1exon9R:

NRG1 Exon 9 (reverse strand)
GCACATTTTACAAGATGGCTTGTCCCAGTGGTGGATGTAGATGTAG

NRG1 Exon 6 (reverse strand)
ATGAAGAAGTATTTGCTCCTTCTGTGGATACTGATATTCTAATGGGAGACT

NRG1 Exon 5 (reverse strand)
CTGAAGACACATATGCTCCTTCAGTTGAGGCTGGCATACCAGTGATGATCT

NRG1 Exon 4 (reverse strand)
CGTTTGATTCCACGATGGTGATATTGGCAGAGGCACTGTCATTTCCTAATTTGCTGATCACTTTGCACATATACT
CTCCAGAATCAGCCAGTGATGCTTTGTTAATGCGAAGTTCTGACTTC

NRG1 Exon 3 (reverse strand)
CCTGGCTTTTTTTGTATCTTGATATTTTGTGGTTTGTTTTTTCGATTCAATTCATTCCCATTCTTGAACCACTTGAA
TCTGAGAGAGGAGTATTCAGAACTGGTTTCACACCGAAGGACTAGTTTGGAACCTGCAGCCGATTCCTGGCTT
TTCATCTCTTTCAATCGGGGAGGCAAGG

TENM4int15 (reverse strand)
CTGATTCCAAGCCTCTTCTCT
GAAAAATCACAGAATCTTGAACAGTTACGTGAGCACTCTGTCCTGTACCACTGGAATC

TENM4 Exon 12 (reverse strand)
CAATGGCAGTGGTGAGAAAGGAAACCACTTCTGACTCCTTTCCGTCATTGTAAAAGCCAAGT

GCCAGATTCCTGAATCCAAATACTGGATGAAGCCTGTCTCATGGCTGGAGGGGGGCACAG
TTCCCCGAGACTGGCGCGGGGTCCCCTCTAGGCTCCGCGCCTCCTGGGTTAGGAGCCTCC
TGCCATCCAGCAGCTCCACAAAGTCAAA

Partial TENM4 Exon 11 (reverse strand)
CTGTGTATGTGAAGGAGGGAGGCCTTTTCTGC
CATAAATGCCAACCAGGGCTGCCTTTCCCAGAGACACATTGAATTTCAGATGCACAGGAT
GGTCTATGAACACTTGAGATCTCCAGAAAGTGCCAGGAGGAATCTTCTGGGAAGCTCGCC
TTCCCACATCAATTTCTCCAGAATCTATGAAACTGTCCTCTGGAAAGAA

Sequencing with NRG1exon4F:

Partial NRG1 Exon4:
GAAATGACAGTGCCTCTGCCAATATCACCATCGTGGAATCAAACG
NRG1 Exon5:
AGATCATCACTGGTATGCCAGCCTCAACTGAAGGAGCATATGTGTCTTCAG
NRG1 Exon6:
AGTCTCCCATTAGAATATCAGTATCCACAGAAGGAGCAAATACTTCTTCAT
NRG1 Exon9:
CTACATCTACATCCACCACTGGGACAAGCCATCTTGTAAAATGTGCGGAGAAGGAGAAAACTTTCTGTGTG
AATGGAGGGGAGTGCTTCATGGTGAAAGACCTTTCAAACCCCTCGAGATACTTGTGCAA
NRG1 Exon11:
GTGCCCAAATGAGTTTACTGGTGATCGCTGCCAAAACTACGTAATGGCCAGCTTCTACA
NRG1 Exon13:
AGGCGGAGGAGCTGTACCAGAAGAGAGTGCTGACCATAACCGGCATCTGCATCGCCCTCCTT
GTGGTCGGCATCATGTGTGTGGTGGCCTACTGCAAAACCAA
NRG1 Exon14:
GAAACAGCGGAAAAAGCTGCATGACCGTCTTCGGCAGAGCCTTCGGTCTGAACGAAACAATATGATGAACAT
TGCCAATGGGCCTCACCATCCTAACCCACCCCCCGAGAATGTCCAGCTGGTGAAT
NRG1 Exon15:
CAATACGTATCTAAAAACGTCATCTCCAGTGAGCATATTGTTGAGAGAGAAGCAGAGACATCCTTTTCCACC
AGTCACTATACTTCCACAGCCCATCACTCCACTACTGTCACCCAGACTCCTAGCCACAG
NRG1 Exon16:
CTGGAGCAACGGACACACTGAAAGCATCCTTTCCGAAAGCCACTCTGTAATCGTGATGTCA
TCCGTAGAAAACAGTAGGCACAGCAGCCCAACTGGGGGCCCAAGAGGACGTCTTAATGGC
ACAGGAGGCCCTCGTGAATGTAACAGCTTCCTCAGGCATGCCAGAGAAACCCCcTGATTC
CTACCGAGACTCTCCTCATAGTGAAAG
Partial NRG1 Exon18:
GTATGTGTCAGCCATGACCACCCCcGGCTCGTATGTCACCTGTAGATTTCCACACGCCA

Sequencing with SP6 primer:

NRG1 Exon18 (reverse strand):
TTTTTCCTGTTTTCTATTTGCAGAACTGCTAAAATAAAATAAA
TTGTTTAATTTAAGGTGGAATACTTTATTATATAAAATAAAGTTTTACAGGTGAATCTAT
GTGTTTATTTAGGTTTTATACAGCAATAGGGTCTTGGTTAGCAATTACACTAGACAGCCT
GGCCTGGATTTCTTCCTGTGTCGAGAAGCGGCCTGCTGGGTTAGTCCTGCTGTCAGCCAG
GCGGAAGGCAGGTGTTGCCTCAAGACTGGCTGCCAGGGGGTTCTGTATGCCCAGGAAAGG
CGTATCTTCACCTACTCTTTCATCTTCTGTTTCACTCTCTGAGTTACTGCTCTGGGAGCT
TGTGTTGCTGTCCACTTCCAATCTGTTAGCAATGTGGCCATTGGGCTTGGTTCTTTTGGC
CCGCCGGCTATTGGCGAGTTTCTTAACAGGCTCTTGGGCTGGCTCGTACTCTTGGGTCGT

TTCATACTCCTCATCCTCCACTATCCTCAAGGGGCTAGCAGGGAGGCTGTTACTGTCATG
CGCGGGGTTGTGGTGGAAGGAGCTGAACTGCTGAGGGTGATGGTCAAACTTCTTCTCCCG
CAGCCTTGGTGGTGTCACGAGAAGTAGAGGTCTCTTCTTCCATGAAGGGGCTGACCGC
CATGGAAGGCATGGACACCGTCATGCTGGACACGGGTGGAGACATTTCCGAAGGGGGCGA
TTTGGGGGAGCTTGGCGTGTGGAAATCTACAGGTGACATACGAGCCGGGGTGGTCATGGC
TGACACATAC
NRG1 Exon16 (reverse strand):
CTTTCACTATGAGGAGAGTCTCGGTAGGAATCAGGGGTTTCTCTGGCATGCCTGAGGAAGCTGTTACATTCAC
GAGGGCCTCCTGTGCCATTAAGACGTCCTC
Continued Exon 16 declining quality sequence:
tTTGGGC CCCCAGTTGGGCTGCTGTGCCTACTGTTTTCTACGGATGACATCACNAATTACAGAGTGG
CTTTCGGAAAGNATGCTTTTCAGTGTGTCCGTTGCTCCAG

SAPS3-TENM4-NRG1 Clone 5
Marked with asterisk in Fig.1. Short isoform matching original cDNA reported by Schaefer et al. (1997). Terminates in extended exon 11 of NRG1.
Sequencing with T7 primer:
vector
GGATCCACTTAGTAACGGCCGCCAGTGTGCTGGA
ATTCTGCAGATATC
SAPS3 exon 1
TGCTTGATACGTCCGCCATTTTGGGCGCTTCGCTGATGGTGTCGGT
GAGCGCGTTTCCCGCCTGAGCGCAACTAGCGGCGGGTCGTGGGCACCTCCAG
TENM4 Exon3:
AAAAGATCCCGCACCATCCTCCAGGATCCAATGGCCTTGGAGAGAGGGCTGCAGGGCCCACGGACATT
GCTGACTCTTCAGAACGTGCTGACATGGAGCCAG
TENM4 Exon4: [T is a C in reference genome]
GTAGACTGAAATTATCATGTGTCCAAATTAAAATTGCATACTTCAAGGATTATTTGAAGGACTATTCTTAGACC
CTTTTAAGAAGATTTAAAGAAAA
TENM4 Exon5:
ACCACTCGGCCCTGAGTGCGGCGAGGACCCTGTTTGTGGATGTGGAGGAGCGCGGGCCGGAGGCCATGGAC
GTGAAGGAGAGGAAGCCTTACCGCTCGCTGACCCGGCGCCGCGACGCCGAGCGCCGCTACACCAGCTCGTCC
GCGGACAGCGAGGAGGGCAAAGCCCCGCAGAAATCGTACAGCTCCAGCGAGACCCTGAAGGCCTACGACCA
GGACGCCCGCCTAGCCTATGGCAGCCGCGTCAAGGACATTGTGCCGCAGGAGGCCGAGGAATTCTGCCGCAC
AG
TENM4 Exon6:
GTGCCAACTTCACCCTGCGGGAGCTGGGGCTGGAAGAAGTAACGCCCCCTCACGGGACCCT
GTACCGGACAGACATTGGCCTCCCCCACTGCGGCTACTCCATGGGGGCTGGCTCTGATGC
CGACATGGAGGCTGACACGGTGCTGTCCCCTGAGCACCCCGTGCGTCTGTGGGGCCGGAG
CACACGGTCAGGGCGCAGCTCCTGCCTGTCCAGCCGGGCCAATTCCAATCTCACACTCA
CCGACACCGAGCATGAAAACACTGAGACTG
Partial TENM4 Exon7:
ATCATCCGGGCGGCCTGC

Sequencing with TENM4exon5F:

TENM4 Exon5:
CCGCCTAGCCTATGGCAGCCGCGTCAAGGACATTGTGCCGCAGGAGGCCGAGGAATTCTGCCGCACAG
TENM4 Exon6:
GTGCCAACTTCACCCTGCGGGAGCTGGGGCTGGAAGAAGTAACGCCCCCTCACGGGACCCTGTACCGGACAG
ACATTGGCCTCCCCCACTGCGGCTACTCCATGGGGGCTGGCTCTGATGCCGACATGGAGGCTGACACGGTGCT

GTCCCCTGAGCACCCCGTGCGTCTGTGGGGCCGGAGCACACGGTCAGGGCGCAGCTCCTGCCTGTCCAGCCG
GGCCAATTCCAATCTCACACTCACCGACACCGAGCATGAAAACACTGAGACTG

TENM4 Exon7:

ATCATCCGGGCGGCCTGCAGAACCACGCGCGGCTCCGGACGCCGCCGCCGCTCTCGCACGCCCACACC
CCCAACCAGCACCACGCGGCCTCCATTAACTCCCTGAACCGGGGCAACTTCACGCCGAGGAGCAACCCCAGCC
CGGCCCCCACGGACCACTCGCTCTCCGGAGAGCCCCCTGCCGGCGGCGCCCAGGAGCCTGCCCACGCCCAGG
AGAACTGGCTGCTCAACAGCAACATCCCCCTGGAGACCAG

TENM4 Exon8:

AAACCTAGGCAAGCAGCCATTCCTAGGGACATTGCAGGACAACCTCATTGAGATGGACATTCTCGGCGCCTCC
CGCCATGATGGGGCTTACAGTGACGG

TENM4 Exon9:

GCACTTCCTCTTCAAGCCTGGAGGCACCTCCCCGCTCTTCTGCACCACATCACCAGGGTACCCACTGACGTCCA
GCACAGTGTACTCTCCTCCGCCCCGACCCCTGCCCCGCAGCACCTTCGCCCGGCCGGCCTTTAACCTCAAGAAG
CCCTCCAAGTACTGTAACTGGAAGTGCGCAGCCCTGAGCGCATCGTCATCTCAGCCACTCTGGTCATCCTGCTG
GCATACTTTGTGG

Partial TENM4 Exon 10:

CCATGCACCTGTTTGGCCTAAACTGGCACCTGCAGCCGATGGAGGGGCAGATGTATGAGAT

Sequencing with NRG1exon9R:

Partial NRG1 Exon 9 (reverse strand):

AGATGGCTTGTCCCAGTGGTGGATGTAGATGTAG

NRG1 Exon6 (reverse strand):

ATGAAGAAGTATTTGCTCCTTCTGTGGATACTGATATTCTAATGGGAGACT

NRG1 Exon 5 (reverse strand):

CTGAAGACACATATGCTCCTTCAGTTGAGGCTGGCATACCAGTGATGATCT

NRG1 Exon4 (reverse strand):

CGTTTGATTCCACGATGGTGATATTGGCAGAGGCACTGTCATTTCCTAATTTGCTGATCACTTTGCACATATACT
CTCCAGAATCAGCCAGTGATGCTTTGTTAATGCGAAGTTCTGACTTC

NRG1 Exon3 (reverse strand):

CCTGGCTTTTTTTGTATCTTGATATTTTGTGGTTTGTTTTTTCGATTCAATTCATTCCCATTCTTGAACCACTTGAA
TCTGAGAGAGGAGTATTCAGAACTGGTTTCACACCGAAGGACTAGTTTGGAACCTGCAGCCGATTCC
TGGCTTTTCATCTCTTTCAATCGGGGAGGCAAGG

TENM4 Exon12 (reverse strand):

CAATGGCAGTGGTGAGAAAGGAAACCACTTCTGACTCCTTTCCGTCATTGTAAAAAGCCAAGTGCCAGATTCC
TGAATCCAAATACTGGATGAAGCCTGTCTCATGGCTGGAGGGGGGCACAGTTCCCCGAGACTGGCGCGGGGT
CCCCTCTAGGCTCCGCGCCTCCTGGGTTAGGAGCCTCCTGCCATCCAGCAGCTCCACAAAGTCAAA

TENM4 Exon11 (reverse strand):

CTGTGTATGTGAAGGAGGGAGGCCTTTTCTGCCATAAATGCCAACCAGGGCTGCC
TTTCCCAGAGACACATTGAATTTCAGATGCACAGGATGGTCTATGAACACTTGAGATCTC
CAGAAAGTGCCAGGAGGAATCTTCTGGGAAGCTCGCCTTCCCACATCAATTTCTCCAGAA
TCTATGAAACTGTCCTCTGGAAAGAAACTACTGGGCTTTC

TENM4 Exon10 (reverse strand):

CTTCTGTGGTTCCTTTGCCTTT

contnuing low quality

NCCTGTCAGGGGTCTCTAAGCCAGTGCCCCCcTGAGGGGTATANGGGAGACGTCGGTTGGCACAGGCCACCT
GcCTGGCTGTGTCCTCCGTGATCTCATACATCTGNCCCCTCCATCG
GCTGCAGGTGCCAGTTTNAGGCCAAACAGGTGCATGG

Sequencing with SP6 primer:

GCTGCTCGAGCGGCCGC

Extended Exon 11(reverse strand):
AGCTCTAGGTGGAATCTGAGGGGAGATGCAGCAACAAGAAAGCA
GCACCAACTGAGCATGCTC
NRG1 Exon11 (reverse strand):
CTATTCAGGCAGAGACAGAAAGGGAGTGGACGTACTGTAGAAGCTGGCCATTACGTAGTTTTGGCAGCGATC
ACCAGTAAACTCATTTGGGCAC
NRG1 Exon9 (reverse strand):
TTGCACAAGTATCTCGAGGGGTTTGAAAGGTCTTTCACCATGAAGCACTCCCCTCCATTCACACAGAAAGTTTT
CTCCTTCTCCGCACATTTTACAAGATGGCTTGTCCCAGTGGTGGATGTAGATGTAG
NRG1 Exon6 (reverse strand):
ATGAAGAAGTATTTGCTCCTTCTGTGGATACTGATATTCTAATGGGAGACT
NRG1 Exon5 (reverse strand):
CTGAAGACACATATGCTCCTTCAGTTGAGGCTGGCATACCAGTGATGATCT
NRG1 Exon4 (reverse strand):
CGTTTGATTCCACGATGGTGATATTGGCAGAGGCACTGTCATTTCCTAATTTGCTGATCACTTTGCACATATACT
CTCCAGAATCAGCCAGTGATGCTTTGTTAATGCGAAGTTCTGACTTC
NRG1 Exon3 (reverse strand):
CCTGGCTTTTTTTGTATCTTGATATTTTGTGGTTTGTTTTTTCGATTCAATTCATTCCCATTCTTGAACCACT
TGAATCTGAGAGAGGAGTATTCAGAACTGGTTTCACACCGAAGGACTAGTTTGGAACCTG
CAGCCGATTCCTGGCTTTTCATCTCTTNCAATCGGGGAGGCAAGG
TENM4 Exon12 (reverse strand):
CAATGGCAGTGGTGAGAAAGGAAACCACTTCTGACTCCTTTCCGTCATTGTAAAAAGCCAAGTGCCAGATTCC
TGAATCCAAATACTGGATGAAGCCTGTCTCATGGCTGGAGGGGGGGCACAGTTCCCCGAGACTGGCGCGGG
GTCCCCTCTAGGCTCCGCGCCTCCTGGGTTAGGAGCCTCCTGCCATCCAGCAGCTCCACAAAGTCAAA
Partial TENM4 Exon11 (reverse strand):
CTGTGTATGTGAAGGAGGGAGGCCTTTTTCTGCCATAAATGCCAACCAGGGCTGCCTTTCCCAGAAACACATT
GAANTTTCAGATGCCCAGGATGGTCTATGAACACTTGANATCTCCAGAAAGTGCCAGGAGGAATCTTCTGGG
